# Upper Gastrointestinal Manifestation of Bezoars and the Etiological Factors: A Literature Review

**DOI:** 10.1155/2019/5698532

**Published:** 2019-07-15

**Authors:** Samiullah Khan, Kui Jiang, Lan-ping Zhu, Iftikhar-ahmad Khan, Kifayat Ullah, Saima Khan, Xin Chen, Bang-mao Wang

**Affiliations:** ^1^Department of Gastroenterology and Hepatology, Tianjin Medical University General Hospital, Tianjin, China; ^2^Department of Endocrinology, Tianjin Medical University General Hospital, Tianjin, China; ^3^Department of Orthopedics, Tianjin Medical University Fourth Central Hospital, Tianjin, China; ^4^Department of Infertility and Reproductive Endocrinology, Tianjin Medical University Central Hospital of Obstetrics & Gynecology, Tianjin, China

## Abstract

A gastric bezoar is a compact mass of indigestible foreign materials that accumulate and consolidate in the stomach; however, it can be found in other sites of the gastrointestinal tract. The causative manner of this condition is complex and multifactorial. The main purpose of the review was to raise awareness among clinicians, particularly gastroenterologists, that patients with certain risk factors or comorbid conditions are predisposed to gastric bezoar formation. Early diagnosis and prompt intervention are crucial to avoid bezoar-induced complications. Upper gastrointestinal endoscopy is the standard diagnostic and therapeutic method for gastric bezoars. However, for large size bezoars, surgical intervention is needed.

## 1. Introduction

Bezoars are congregations or compact masses that formed by the accumulation of matter, especially nonedible materials, including high-fiber vegetable diet, hair, and certain pharmaceutical agents. They are found more frequently in the stomach in patients with normal or abnormal gastric function or in patients with poor gastric peristalsis resulting in delayed gastric draining and other associated disorders [1, 2].

The majority of gastric bezoars are found to be present in adolescents and young ladies with a history of pica, predominantly psychiatric disorders. In contrast to adults, the majority of gastric bezoars are associated with gastroparesis, anatomical abnormalities, and former gastric surgeries that reduced gastric motility and ultimately resulting in delayed stomach emptying [1].

The most common clinical presenting symptoms in patients with gastric bezoars include nausea and vomiting, epigastric pain, dyspepsia, and weight loss [1, 3]. They can also be discovered accidentally in asymptomatic patients who undergo upper gastrointestinal (GI) endoscopic evaluation for other indications.

### 1.1. Etiological Factors and Classifications

Bezoars occur most commonly in people with certain risk factors (Table 1) [4–14] or in patients with coexisting medical disorders (Table 2) [2, 12, 14–44].

Bezoars are categorized according to the following materials that form them.

1. Phytobezoars or diospyrobezoar: composed of indigestible fruit or vegetable content
2. Trichobezoars: composed of hair
3. Lactobezoars: composed of milk products
4. Pharmacobezoars: composed of tablets and medications

Hypothetically, the partially digested and undigested materials accompanied by gastric mucus can be a source of gastric bezoar.

## 2. Risk Factors

### 2.1. High Fiber Diet

Diets with high-fiber content (vegetables and fruits, i.e., cellulose) are more common in regions where cultures/beliefs play a key role in consumption [4]. A high-fiber diet has many benefits and is being suggested by health care institutions. Though this suggestion is appropriate for wider consumers and especially the aged population [45], the people with previous gastric surgeries should avoid high-fiber intake because they are more likely to form gastric phytobezoars. These fibers are found in fruits and vegetables including celery, pumpkin, green beans, prunes, raisins, leeks, beets, and sunflower seed shells that are merged into a mass and most often contribute to the development of gastric bezoar [3]. A specific kind of phytobezoar named a diospyrobezoar is made from unripened persimmons, coconuts, and jujubes [1, 5]. A gastric bezoar has also been reported in a patient taking vegetable-derived oil touted to contain lecithin for health purposes in lowering cholesterol levels and improving memory [46].

### 2.2. Undigested Milk Products

A gastric lactobezoar is a mass composed of a specific form of inspissated milk and mucus components [6]. This type of bezoar is commonly discovered in premature kids receiving formula diets [8]. The pathogenesis is usually complex, involving both exogenous and endogenous risk factors (i.e., synthetic milk, feeding methods, dehydration, premature birth, low birth weight, and insufficient activity and capacity of the GI tract) [6, 7]. Rarely, gastric bezoars may develop in pediatric patients with failure to thrive and iron deficiency anemia due to malnutrition [43]. Moreover, recent advances in artificial milk conformation, mother's education, and improvements in premature newborn management dramatically affected the incidence of gastric lactobezoar.

### 2.3. Pharmaceutical Agents

Pharmacobezoars are characterized by aggregations of medicines that do not properly liquefy in the GI tract and can be found in patients taking a pharmaceutical agent, tablets or somewhat liquid masses of drugs; they are usually found following an overdose of medications or in a suicidal attempt [9]. The most frequently involved medication in this entity is bulk-forming hygroscopic laxatives, e.g., perdiem and psyllium preparations, guar gum [6]. Because of the advancement of technology and time delivery-facilitated drug tablets/capsules to be slowly dissolved and gradually release active ingredients of the medication, extended-release medicines, e.g., nifedipine and verapamil, are coated with cellulose acetate; cellulose acetate may amass and lead to the progression of gastric bezoar [6]. Moreover, aluminum hydroxide gel, enteric-coated aspirin, sucralfate, cholestyramine, enteral feeding formulas, mesalamine pills, and meprobamate appear to contribute to the development of pharmacobezoars [47, 48]. Furthermore, a case by Croitoru et al. [10] reported a sodium polystyrene sulfonate gastric bezoar in a patient who mechanically ventilated after cardiopulmonary resuscitation secondary to pericarditis, primary lung cancer, and kidney failure with concomitant hyperkalemia.

### 2.4. Pica Ingestion

Pica consumption is closely linked to buildup gastric mass characterized by mainly nonnutritious materials, such as ice, pagophagia; paper, papyrophagia; drywall or paint; metal, metallophagia; stones, lithophagia; soil, geophagia; glass, hyalophagia; feces, coprophagia; and chalk. Pica consumption is most frequently found in pregnant women, small children, and those with developmental abnormalities, such as autism [11]. Children ingesting painted plaster may suffer brain damage and learning disabilities from lead poisoning. Furthermore, there is a high risk of GI obstruction or tearing in the stomach. Pica has recently been reported in patients with postbariatric surgery, who presented with pagophagia [49].

### 2.5. Impaired Mastication

Mastication is a multifactorial semiautonomic sensory motor pathway by which food content is converted into a bolus throughout the course of intraoral manipulation. Influencing factors involve dental status, active adaptation in conducting mastication during bolus formation and properties amalgamation of a bolus which may increase the possibility of GI diseases and reduce gut absorption. Mastication efficacy in denture wearers and dentate subjects is vastly different. In denture wearers, the mastication is known to be highly impaired during bolus formation. In addition to abnormal chewing behaviors and gastric motility, delay gastric emptying occurs due to large fragmented gastric bolus and consequently multiple gastric anomalies [12, 13].

### 2.6. Inadequate Fluid Intake

Fluids play a critical role in the regularity and the avoidance of GI disorders. Dietary fluid intake and renal excretion regulate total body sodium content. Inadequate fluid intake causes low blood pressure, constipation, kidney disease, electrolyte imbalance, mental changes, and dry stomach. Adequate fluids provide the source for the production of mucus in the GI tract and keep things lubricated and moistened, and thereby, the food bolus and stool can easily move through the GI tract and thus prevented GI disorders [14]. In addition, aged people and the people who work in hot climates are susceptible to dehydration and malnourishment due to age factors, economic status, and environmental factors.

### 2.7. Honeycomb Ingestion

Recently, honeycomb consumptions are widely used for various health purposes such as heart diseases, liver diseases, and metabolic disorder. However, ingesting a huge quantity of honeycomb may cause GI obstruction and life-threatening consequences. Moreover, Katsinelos et al. [14] reported a patient with irritable bowel syndrome who consumed a large quantity of honeycomb for relieving the symptoms and eventually developed a giant gastric bezoar.

## 3. Comorbid Conditions

### 3.1. Coexisting Medical Disorders

#### 3.1.1. Psychiatric Disorders

Trichobezoar commonly appears in patients with a history of Rapunzel syndrome. In this condition, patients have significant psychological or behavioral abnormalities most commonly found in females and can be associated with trichotillomania and trichotillophagia (urge to pullout one's own hair) combined with trichophagia [2, 17]. Rarely, recurrent trichobezoar may link with animals' feet stew with skin and hair intact [15]. Gastric bezoars with anorexia nervosa, bulimia nervosa [16–18], and sickle cell disease [19] have also been reported in this entity.

#### 3.1.2. Gastrointestinal Amyloidosis

Amyloidosis is a condition caused by deposition of unsolvable abnormal (misfolded protein) amyloid fibrils that modify the normal function of organs and tissues [20]. The small bowel is the most common site for amyloid deposits [21]. Numerous endoscopic features of gastric amyloidosis are nonspecific. Findings include erosions, ulcerations, thickened gastric folds, friability, edema, and submucosal hematoma [50]. The delay of gastric emptying can be the result of several causes. However, amyloid light-chain amyloidosis and amyloid A amyloidosis subtype [21] can cause abnormal GI peristalsis that consequently delayed emptying of food from the stomach and leads to the formation of bezoar [20].

Certain comorbid conditions [11] such as diabetes mellitus, cystic fibrosis, Guillain–Barre syndrome, Bouveret's syndrome, hypothyroidism, renal failure, scleroderma, myotonic dystrophy, Ménétrier's disease, multiple myeloma, and hypochlorhydria or achlorhydria have been associated with a higher risk of bezoar formation. (1) Diabetes mellitus is a disorder that causes gastroparesis as a specific complication of diabetes which does not seem to raise the mortality rate. The series of gastric motor irregularities among diabetic patients like irregular distribution of gastric food, a decreased incidence of the antral element that induces antral hypomotility, antral dilatation, fasting, postprandial hypomotility, electrical dysrhythmias, reduced fundic tone, and hyperglycemia can delay gastric emptying [44]. (2) Cystic fibrosis is a hereditary condition that causes intense damage to the lungs, gastrointestinal system (malabsorption), and other organs in the body. Cystic fibrosis potentially dysfunction exocrine gland cells, including mucus-producing cells, sweat, and cells of digestive enzymes. According to Ong et al. [22], these secreted fluids of exocrine glands are generally thin and greasy. But in people with cystic fibrosis, a faulty gene cystic fibrosis transmembrane conductance regulator protein causes the secretions to become sticky, thick, and block lumens. (3) Guillain–Barre syndrome is however rarely associated with a gastric mass and characterized by an acute inflammatory demyelinating polyneuropathy, affecting the peripheral nervous system which leads to weakness and loss of tendon reflexes, dysphagia, difficulty in chewing, and loss of sphincter functions [23]. (4) Bouveret's syndrome is a very rare form of gallstone ileus caused by the passage and impaction of a large gallstone which passes into the duodenal bulb through a cholecystogastric or cholecystoduodenal fistula and ultimately blocks gastric outflow [24, 25]. Gastric-outlet-obstruction can be due to bacterial infection or gastric wall abscess after cholecystitis [26]. (5) Hypothyroidism, myxoedema or underactive thyroid, is mostly seen in women and is believed to cause gastric bezoar. It is a condition causing slowdown metabolism, GI upset, constipation, etc. [27]. (6) Renal failure is one of the leading causes of delayed gastric emptying and gastric stasis, especially in uremic patients and uremic neuropathy that are so common in these patients [28, 29]. (7) Scleroderma is a prolonged autoimmune disease that is usually associated with abnormal GI motility more commonly in patients with diffuse or limited scleroderma which causes malabsorption, weight loss, severe malnutrition, and delayed gastric emptying in the absence of a mechanical obstruction [30, 31]. (8) Myotonic dystrophy or muscular dystrophy is known to cause GI motility disorder such as edema, atrophy, and fibrosis of smooth muscles of the GI tract. The most common is the Duchenne muscular dystrophy. It is a long-term genetic disorder that affects the function muscles characterized by progressive destruction of striated muscular fibers that may often contract and/or unable to relax [32, 33]. (9-10) Rarely, intragastric bezoar may be associated with multiple myeloma [51] and Ménétrier's disease [34]. Ménétrier's disease is a rare condition characterized by gyriform or nodular enlargement of gastric mucosal folds and protein-losing hypertrophic gastroenteropathy. (11) Hypochlorhydria [14] or achlorhydria is a condition of a mild or complete absence of hydrochloric acid in gastric secretions of the stomach and other digestive organs due to dietary factors or medical interventions, respectively. This results in impaired digestion and numerous other effects on the GI tract. Moreover, hypomotility and hyposecretion are the two most significant factors in gastric bezoar formation.

### 3.2. Anatomic Abnormalities

#### 3.2.1. Gastric Diverticula

A gastric diverticulum is a rare cause of gastric bezoar when a bulk of undigested food remnant expelled from the diverticula of size (1-10 cm). It can be categorized into congenital type and acquired type. The congenital type being more common and less involved in gastric mass formation compared to acquired type is mostly found in the posterior wall of the fundus and account for about 70%. The false diverticula are usually located in the gastric antrum and greater curvature with a contextual history of chronic GI diseases, such as peptic ulcer, pancreatitis, malignancy [52], surgical management with amputation, and gastric segmental resection [35, 36].

#### 3.2.2. Pyloric Stenosis

Pyloric stenosis is a tightening of the pyloric canal most frequently found in infants with a cesarean section or preterm birth [53]. The etiology of pyloric stenosis is complex, with some genetic and some environmental factors. Adults with pyloric stenosis may be due to the idiopathic hypertrophic pylorus [37] or related to underlying gastric pathology such as recurrent peptic ulcers, malignancy, and hypertrophic gastritis that weakens gastric emptying into the duodenum; as a result, all consumed foodstuff stuck in the stomach due to the pyloric obstruction and developed gastric mass [48]. Pyloric obstruction can also be a result of Bouveret's syndrome [24] and bacterial infection of the gastric wall or gastric wall abscess after cholecystitis [26]. Endoscopic submucosal dissection of the pyloric ring has also been found to be a risk factor for pyloric stenosis [38].

Rarely, gastric bezoars formed when gallstone migrated to the stomach through a cholecystogastric fistula [39] or cholecystoduodenal fistula after endoscopic retrograde cholangiopancreatography [12]. In most cases, the gallstone enters the duodenum through a cholecystoduodenal fistula followed by retrograde migration to the stomach. Small stones are generally eliminated via the stools, and stones measuring more than 2.5 cm are likely to cause obstruction [54]. The most common clinical manifestation is an acute obstruction, either at the duodenum bulb, causing pyloric obstruction, or at the ileum, causing gallstone ileus. Diabetic diathesis might be the major risk factor accountable for producing the pathologic derangement of the gallbladder and stomach and earlier history of gastroparesis, which led to the formation of bezoar and severe complications [39].

### 3.3. Gastric Dysmotility

#### 3.3.1. Gastroparesis

Gastroparesis or gastric stasis is a disorder that affects gastric muscle activity, and consequently, foodstuff rests in the stomach for a prolonged time [41]. The causative factor of gastric stasis is usually unknown. However, the gastric motor defect may result from autonomic neuropathy, enteric neuropathy; defective interstitial cells of Cajal, diabetes mellitus, develop gastroparesis or idiopathic gastroparesis [40]. Moreover, postoperative gastroparesis is often caused by damage to the vagus nerve.

#### 3.3.2. Previous Gastric Surgeries

The majority of gastric bezoars develop in patients with previous gastric surgeries such as Laparoscopic adjustable gastric banding [42, 43] and Roux-en-Y gastric bypass [55, 56]. Bezoars can develop months to years postoperatively. People, who undergo surgical procedures for bariatric surgery, and particularly partial gastrectomy for gastric cancer are prone to form gastric bezoars due to reduced gastric motility, loss of antral-pyloric function, hypoacidity, and rarely vagotomy that are the major causes of gastric stasis [14, 57].

## 4. Diagnostic Workup

**Figure 1:**
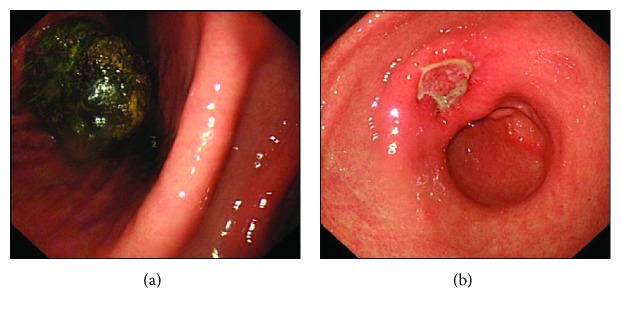
(a) Upper GI endoscopy showing a giant 7 × 5 cm diameter gastric diospyrobezoar. (b) Upper GI endoscopy showing a necrotic pressure ulcer of size 0.8 × 0.5 (white coated).

Gastric bezoars are usually asymptomatic. They are rarely suspected by referring clinicians except in psychiatric patients. They often cause ulceration due to pressure necrosis, pyloric obstruction, peritonitis, and rarely perforation [2, 3, 58] (Figures 1(a) and 1(b)). Therefore, prompt diagnosis and early management of gastric bezoars are essential. A summary of case studies regarding gastric bezoars is presented in Table 3.

An abdominal examination has limited the efficacy in identifying gastric masses; though, sometimes on abdominal palpation intragastric mass or halitosis from the putrefying items can be found. However, these observations are not definitive and much harder to differentiate.

Upper GI series is the first step in diagnosis gastric bezoar if suspected. Appearance on CT is a mass-like filling defect with various composition-dependent characteristics. Trichobezoars often have a lamellated appearance. The gold standard for imaging is direct visualization with upper GI endoscopy for both diagnostic and therapeutic purposes [1, 14].

## 5. Management

Gastric bezoars can be managed either medicinally, endoscopic, or surgically. Bezoars with small size may pass via the GI tract freely on their own. In the management of gastric bezoars, there are three most common approaches which mostly focus on dissolution or eliminating bezoars. (1) Enzymatic treatment (Coca-Cola irrigations, gastroprokinetic agents, and enzymes cellulose) [4, 5, 18]. (2) Endoscopic management as the mainstream treatment includes (biopsy and alligator forceps, lithotripters, needle cutter, snares of polypectomy, and lithotripsy with Nd:YAG laser-ignited mini-explosive procedure) [4, 59]. (3) However, surgical management is the best technique for bigger ones. Recently, a laparoscopic procedure with Alexis wound retractor was effectively used in the management of bezoars [2, 4, 60]. More recently, holmium:YAG (Ho:YAG) laser lithotripsy for giant bezoar and a laparoscopic technique with endobag in the stomach to prevent bezoar spillage have shown promising results [59]. Traditional Chinese medicine purgative has also shown effectiveness in the dissolution of giant gastric bezoar and associated gastric lesions [61]. Furthermore, psychiatric treatment and dietetic instruction are suggested.

## 6. Conclusions

Gastric bezoars most frequently occur in patients with certain risk factors including psychiatric conditions, anatomic anomalies, and weakened gastric motility or in patients with coexisting medical conditions. Early diagnosis and appropriate treatment strategy are essential to prevent bezoar-induced complications. Upper GI endoscopy is a safe and effective procedure for diagnostic and therapeutic purposes of gastric bezoars. Besides, careful endoscopic surveillance should be carried out if the bezoars recur repeatedly, especially in patients with anatomical abnormalities or previous gastric surgeries. There could be a number of other contributing factors that can lead to gastric bezoar but have not yet been known to the clinicians. However, further studies are required to address this issue.

## Figures and Tables

**Table 1:** Most common risk factors associated with gastric bezoars.

	Fibers rich diet	Milk products	Medications	Pica consumption	Mastication disorders	Insufficient fluid intake	Honeycomb consumption
Risk factors	Vegetarians	Synthetic milk	Overdose medicines	Nonnutritive constituents	Dental status	Elder people	Patients with large quantity of honeycomb ingestion for multiple health benefits
	Fiber-rich fruits	Feeding method	Medication for suicidal attempt	Pregnant women and small children	Abnormal mastication	Labors in hot climate	
	Patient with partial gastrectomy on a high-fiber diet	Premature birth	Bulk-forming agents	Patients with autism	Denture wearers	Inadequate fluid ingestion	
		Failure to thrive	Extended-release medications	Patients with bariatric surgery		Kidney disease	
		Anemic children					

**Table 2:** Most common comorbid conditions associated with gastric bezoars.

	Medical disorders	Anatomic abnormalities	Gastric motility disorders
Comorbid conditions	Rapunzel syndrome Anorexia nervosa & bulimia nervosa Sickle cell & gastrointestinal amyloidosis Diabetes mellitus & cystic fibrosis Guillain–Barre syndrome & Bouveret's syndrome Hypothyroidism & renal failure Scleroderma & myotonic dystrophy Ménétrier's disease Hypochlorhydria or achlorhydria	Gastric diverticula Gastric outlet obstruction Pyloric stenosis Cholecystogastric fistula Cholecystoduodenal fistula	Gastroparesis Diabetic gastroparesis Idiopathic gastroparesis Postsurgical gastroparesis Previous gastric surgeries

**Table 3:** A summary table with case studies regarding gastric bezoars.

Case no. A/G [ref no.]	History/previous operation	Symptoms	Clinical findings	Locations of bezoar in the stomach	Size of bezoar (cm)	Associated gastric lesions	Composition of the bezoar	Management	Complications
(1) 49/M [1]	Habitual jujubes ingestion	Epigastric pain Nausea and vomiting Gastric reflux	Anemic Abdominal tenderness	Body	8 × 5 cm	Necrotic ulcer	Jujubes (diospyrobezoar)	Coca-Cola Lithotripsy	None
(2) 18/F [2]	Trichophagia (Rapunzel syndrome)	Acute abdominal pain Vomiting	Weight loss	Full-length	120 cm	Ulcer	Hair (trichobezoar)	Laparotomy	Gastric perforation
(3) 47/M [3]	6-month	Epigastric pain	Weight loss	Body	9 × 4 cm	None	Phloem fibers Raw stinging nettle (phytobezoar)	Laparotomy	None
(4) 76/M [4]	Arterial hypertension	Dyspepsia Epigastric pain	None	Body	10 cm	Ulcer	Vegetable fibers (phytobezoar)	Endoscopic (polypectomy snare)	None
(5) M [46]	None	Abdominal pain Early satiety	Weight loss	Body	N/A	None	Fatty acids and lecithin (phytobezoar)	Surgical removal	None
(6) 96 cases [7]	Prematurity Low birth weight	Abdominal distension Vomiting Diarrhea	Palpable abdominal mass	N/A	N/A	None	High casein content 54.2%, medium chain triglycerides 54.2% Caloric density 65.6% (lactobezoars)	Cessation of oral feedings administration of intravenous fluids Gastric lavage surgery	Perforations (7 patients)
(7) 44/F [9]	Anxiety disorder	Semiconscious Fast breathing	Potassium overdose (hyperkalemia) Bp-89/59 mmHg Pulse 82/min, resp. 20/min	Gastric fundus	N/A	None	Extended-release potassium chloride (pharmacobezoar)	Whole bowel irrigation using polyethylene glycol (NG tube) Upper GI endoscopic removal of pharmacobezoar	None
(8) 60/F [47]	Open cholecystectomy and choledicholithotomy	Epigastric pain Vomiting	Mildly anemic Dehydrated Tachycardia Epigastric tenderness	Pyloric canal	N/A	None	Aluminum hydroxide tablets (pharmacobezoar)	Endoscopic removal using biopsy forceps and Dormia basket	None
(9) 58/M [48]	3-month Suspected Crohn's disease	Abdominal pain Vomiting	Circumferential wall thickening of pylorus	Pylorus	N/A	Gastritis noncaseating epithelioid Multiple hyperplastic polyps	Mesalamine pills (pharmacobezoar)	Laparoscopic gastrojejunostomy	Gastric outlet obstruction
(10) 54/M [10]	Primary lung cancer (metastatic) Mechanically ventilated	Hyperkalemia	Constrictive effusive metastatic pericarditis kidney failure	Body	13 × 6 × 7 cm	None	Sodium polystyrene sulfonate (pharmacobezoar)	Postmortem	Expired
(11) 7/M [11]	Pica	Abdominal pain Vomiting	Abdominal tenderness guarding	Full-length gastric bezoar	13 × 11 cm	None	Wooden bezoar	Laparoscopic Psychiatric evaluation	Gastric perforation
(12) 53/F [49]	Pica (anxiety, depression) Roux-en-Y gastric bypass	Severe personality disorders Vomiting Constipation	Mild abdominal distention	N/A	2.5 × 1 × 0.8 cm	None	Cardboard and newspaper	Endoscopic removal Psychiatric evaluation	None
(13) 69.4 ± 5.7 M/F [13]	Denture wearers Impaired mastication (8 male/6 female)	Not mentioned	Muscle bursts were longer = lower muscle work Muscle burst decreased significantly for denture wearers Longer chewing duration Food boli were less disorganized	N/A	N/A	N/A	Chewing of paraffin and meat	N/A	Impaired chewing in complete denture wearers modifies the dynamics of meat bezoar formation due to large fragmented bolus
(14) 44/F [14]	Irritable bowel syndrome Consumption of large quantities of honeycomb for health benefits	Epigastric pain Nausea	None	Body	N/A	N/A	Honeycomb	Endoscopic removal 100 ml of hydrogen peroxide Modified and conventional needle-knife Snares and baskets	None
(15) 69/F [12]	Cholelithiasis Choledocholithiasis	Right-sided upper abdominal pain Nausea and vomiting	Multiple biliary stones in the common bile duct	Pylorus and duodenal bulb	N/A	N/A	Gallstones and indigestible material	Proton pump inhibitor and cola drink	None
(16) 14/F [17]	Anorexia nervosa Thalassemia trait and growth hormone replacement. Trichotillomania	Nausea and vomiting	Weight loss Nontender, large, firm, left upper quadrant mass	Full-length (entire stomach and duodenum)	N/A	None	Hair (trichobezoar)	Laparotomy	None
(17) 45/F [15]	Habitual consumption of cows' feet stew with hair and skin intact. Previous history of gastric bezoar via laparotomy	Dysphagia Abdominal distension Abdominal pain Shortness of breath Generalized weakness	Microcytic anemia Malnourished CT = large gastric bezoar	Lesser curvature	2.42 kg	Ulcer at the lesser curvature	Mass of hair Leathery skin and altered food (trichobezoar)	Laparotomy Gastrotomy	None
(18) 19/F [16]	Anorexia nervosa Binge-purge Hematemesis	Nausea and vomiting Constipation	Weight loss Parotid hypertrophy bilaterally	Vomited a cylindrical bezoar from the stomach	4 cm	Possible erosions or ulcer	Debris and birefringent Foreign material Vegetable matter	Conservative treatment	N/A
(19) 21/F [18]	Bulimia nervosa Binge eating episodes	Abdominal pain Nausea Retching	Afebrile, normotensive with mild tachycardia Distended abdomen Weight loss	Greater curvature overlying the pylorus	30.9 × 16.1 cm	None	Food matter	Coca-Cola Metoclopramide Endoscopic Psychotherapy	None
(20) 3/F [19]	Sickle cell disease	Upper abdominal pain Nonbilious emesis Anorexia	Large intra-abdominal mass epigastric tenderness Hemoglobin 9.6 g/dL Leukocyte 20.4 × 10^3^/*μ*L Polymorphonuclear leukocyte 69% Platelet 254,000/*μ*L	Stomach extended to the duodenum	12 × 6 × 4 cm	N/A	Trichobezoar	Laparotomy Gastrotomy	None
(21) 62/F [51]	Multiple myeloma	Epigastric pain Vomiting Weight loss Fatigue	Elevated IgG of 49.2 g/L Low IgM and IgA levels IgG Lambda paraprotein 35 g/L Lambda Bence-Jones protein in the urine, elevated *β*_2_-microglobulin 5.50 mg/mL	Body extended pylorus	N/A	Mild focal intestinal metaplasia and glandular atrophy	Phytobezoar	Coca-Cola pancreatic enzyme supplementation	Expired in 1 month
(22) 42/F [39]	Hypertension Type 2 diabetes mellitus Peripheral neuropathy Gastroparesis	Nausea and vomiting Abdominal pain Fever	Obese, epigastric tenderness Significant distress Abdominal distension Hypoactive bowel sounds	Antrum	5.5 × 3.5 cm	N/A	Cholesterol gallstone induced bezoar	Laparotomy Gastrotomy	None
(23) 34/F [42]	Laparoscopic adjustable gastric banding	Epigastric fullness Nausea and vomiting	Obese BMI 37 kg/m2	In eccentric pouch dilatation	N/A	N/A	Bezoar	Liquid diet Laparoscopy	Anterolateral slippage of the band
(24) 48/M [43]	Laparoscopic adjustable gastric banding	Dysphagia	N/A	Body	N/A	Erosions	Phytobezoar	Papain (1 week)	None
(25) 70/M [54]	Cholecystogastric fistula	Painful lump in the right hypochondriac region with fever and anorexia	CT revealed fistula between the gallbladder and gastric antrum.	Antrum	9 × 5 × 5 cm	Fistulous opening in the prepyloric region	Gallstone bezoar (cholesterol and calcium oxalate)	Laparotomy	None
(26) 63/F [56]	Roux-en-Y gastric bypass	Abdominal distention Nausea and vomiting	Morbid obese (body mass index 49.5 kg/m^2^) 14 months postsurgery BMI 28 kg/m^2^	Gastric pouch	5 cm	None	Persimmon Vegetables	Endoscopic Biopsy snare	None
(27) 65/M [58]	Chestnuts consumption	Abdominal pain	Abdominal CT indicated gastric perforation	Lesser curvature	N/A	Ulcer	Tannin Chestnut bezoars	Surgery Coca-Cola	Gastric perforation
(28) 73M/58F [59]	(2 cases) (1) Billroth I partial gastrectomy for gastric cancer. (2) Laparoscopic adjustable gastric banding	N/A	Cancer Obesity	Proximal gastric pouch	10 cm 8 cm	N/A	Phytobezoar	200 micron laser fiber and 550 micron laser fiber (Ho:YAG laser)	None
(29) 62/F [61]	Acute gastritis and gallstones	Epigastric pain Nausea and vomiting Hiccups Heartburn Dark loose stools	Abdominal tenderness Positive Murphy sign Hyperactive bowel sounds Pale tongue Occult blood in the vomit	Body	N/A	Gastric angle with multiple lesions Bleeding Gastric ulcers Venous aneurysm	Bezoar	Chinese medicine purgative combined with pantoprazole sodium intravenous infusion, 40 mg each time, twice a day for 5 days	None

A/G: age/gender; M: male; F: female; NA: not available; cm: centimeter.

## Abbreviations

GI: Gastrointestinal
Ho:YAG: Holmium:YAG.

## References

[1] Khan S., Khan I. A., Ullah K. (2018). Etiological aspects of intragastric bezoars and its associations to the gastric function implications: a case report and a literature review. *Medicine*.

[2] Parakh J. S., McAvoy A., Corless D. J. (2016). Rapunzel syndrome resulting in gastric perforation. *Annals of the Royal College of Surgeons of England*.

[3] Gachabayov M., Abdullaev A., Mityushin P., Gilyazov T. (2016). Each worm to his taste: some prefer to eat nettles – a giant gastric phytobezoar. *Clinical Case Reports*.

[4] Ugenti I., Travaglio E., Lagouvardou E., Caputi Iambrenghi O., Martines G. (2017). Successful endoscopic treatment of gastric phytobezoar: a case report. *International Journal of Surgery Case Reports*.

[5] Ladas S. D., Kamberoglou D., Karamanolis G., Vlachogiannakos J., Zouboulis-Vafiadis I. (2013). Systematic review: Coca-Cola can effectively dissolve gastric phytobezoars as a first-line treatment. *Alimentary Pharmacology & Therapeutics*.

[6] Iwamuro M., Okada H., Matsueda K. (2015). Review of the diagnosis and management of gastrointestinal bezoars. *World Journal of Gastrointestinal Endoscopy*.

[7] Heinz-Erian P., Gassner I., Klein-Franke A. (2012). Gastric lactobezoar - a rare disorder?. *Orphanet Journal of Rare Diseases*.

[8] Castro L., Berenguer A., Pilar C., Goncalves R., Nunes J. L. (2014). Recurrent gastric lactobezoar in an infant. *Oxford Medical Case Reports*.

[9] Briggs A. L., Deal L. L. (2014). Endoscopic removal of pharmacobezoar in case of intentional potassium overdose. *The Journal of Emergency Medicine*.

[10] Croitoru M., Shouval A., Chepurov D., Katz Y. (2015). Giant intragastric sodium polystyrene sulfonate bezoar. *Kidney International*.

[11] Karnik P. P., Dave N. M., Garasia M. (2016). Large gastric wood bezoar: anesthesia implications. *Journal of Anaesthesiology Clinical Pharmacology*.

[12] Sarikaya M., Koçak E., Köklü S., Akbal E. (2012). Acute gastric obstruction resulting from gallstone-induced bezoar. *The American Surgeon*.

[13] Yven C., Bonnet L., Cormier D., Monier S., Mioche L. (2006). Impaired mastication modifies the dynamics of bolus formation. *European Journal of Oral Sciences*.

[14] Katsinelos P., Pilpilidis I., Chatzimavroudis G. (2009). Huge gastric bezoar caused by honeycomb, an unusual complication of health faddism: a case report. *Cases Journal*.

[15] Kiernan M. F., Kamat S., Olagbaiye F. (2012). Cows-feet soup: a rare cause of recurrent trichobezoar. *Case Reports*.

[16] Laird Birmingham C., Cardew S., Gritzner S. (2007). Gastric bezoar in anorexia nervosa. *Eating and Weight Disorders - Studies on Anorexia, Bulimia and Obesity*.

[17] Saldanha N. E., Meisel J. A., Prince J. M., Feinstein R., Fisher M. (2015). Delayed diagnosis of trichobezoar in a patient with presumed anorexia nervosa. *International Journal of Adolescent Medicine and Health*.

[18] Fazio R. M., Shah P., Soe E., Iswara K., Chen I. (2016). Gastric bezoar causing massive gastric distention and functional outlet obstruction in a patient with bulimia nervosa. *Journal of Medical Cases*.

[19] Sciarretta J. D., Bond S. J. (2011). Gastric trichobezoar: abdominal mass in a child with sickle cell disease. *Pediatric Emergency Care*.

[20] Lee A. S. Y., Lee D. Z. Q., Vasanwala F. F. (2016). Amyloid light-chain amyloidosis presenting as abdominal bloating: a case report. *Journal of Medical Case Reports*.

[21] Stofer F., Barretto M. F., Gouvea A. L. (2016). A rare case of ascites due to peritoneal amyloidosis. *American Journal of Case Reports*.

[22] Ong T., Marshall S. G., Karczeski B. A., Sternen D. L., Cheng E., Cutting G. R., Pagon R. A., Adam M. P., Ardinger H. H. (1993). Cystic Fibrosis and Congenital Absence of the Vas Deferens. *GeneReviews(R)*.

[23] Colantuoni M., Matano E., Alfieri S., De Placido S., Carlomagno C. (2010). Guillain-Barre syndrome associated with gastric cancer: paraneoplastic syndrome or immunological disorder?. *World Journal of Oncology*.

[24] Qamrul Arfin S. M., Haqqi S. A., Shaikh H., Wakani A. J. (2012). Bouveret’s syndrome: successful endoscopic treatment of gastric outlet obstruction caused by an impacted gallstone. *Journal of the College of Physicians and Surgeons Pakistan*.

[25] Foets T. C., Weusten B. L., van Es H. W., Boerma D. (2014). An 84 year old man with gastric outlet obstruction. *Nederlands Tijdschrift voor Geneeskunde*.

[26] Soga K., Kassai K., Itani K., Yagi N., Naito Y., Itoh Y. (2014). Gastric outlet obstruction induced by a gastric wall abscess after cholecystitis. *Internal Medicine*.

[27] Kaplan L. R. (1980). Hypothyroidism presenting as a gastric phytobezoar. *American Journal of Gastroenterology*.

[28] Hirata E. S., Mesquita M. A., Alves Filho G., Terra C. H. (2007). O esvaziamento gástrico e a insuficiência renal crônica. *Revista Brasileira de Anestesiologia*.

[29] Shirazian S., Radhakrishnan J. (2010). Gastrointestinal disorders and renal failure: exploring the connection. *Nature Reviews. Nephrology*.

[30] Piskorz M. M., Rank G., Velazquez Espeche M. (2015). Usefulness of gastric emptying scintigraphy for the evaluation and management of scleroderma related gastroparesis. *Acta Gastroenterologica Latinoamericana*.

[31] Nagaraja V., McMahan Z. H., Getzug T., Khanna D. (2015). Management of gastrointestinal involvement in scleroderma. *Current Treatment Options in Rheumatology*.

[32] Lo Cascio C. M., Goetze O., Latshang T. D., Bluemel S., Frauenfelder T., Bloch K. E. (2016). Gastrointestinal dysfunction in patients with Duchenne muscular dystrophy. *PLoS One*.

[33] Fois A. (1997). Gastrointestinal disorders in muscular dystrophies. *Journal of Pediatric Gastroenterology and Nutrition*.

[34] Anandpara K. M., Aswani Y., Hira P. (2015). An unusual association of Menetrier’s disease with a gastric bezoar. *Case Reports*.

[35] Podda M., Atzeni J., Messina Campanella A., Saba A., Pisanu A. (2016). Syncope with surprise: an unexpected finding of huge gastric diverticulum. *Case Reports in Surgery*.

[36] Moy B. T., Beery R. M. M., Birk J. W. (2016). Gastric diverticulum: an unusual endoscopic finding. *ACG Case Reports Journal*.

[37] Holder J., Zinn D., Samin A. (2017). Adult-onset idiopathic hypertrophic pyloric stenosis associated with osteoglophonic dysplasia and HIV: case report and review of literature. *Ultrasound Quarterly*.

[38] Lee J. U., Park M. S., Yun S. H. (2016). Risk factors and management for pyloric stenosis occurred after endoscopic submucosal dissection adjacent to pylorus. *Medicine*.

[39] Tadros G. M., Draganescu J. M., Clarke L. E., Albornoz A. M. (2002). Intragastric gallstone-induced bezoar: an unusual cause of acute gastric outlet obstruction. *Southern Medical Journal*.

[40] Mohammad M. K., Pepper D. J., Kedar A. (2016). Measures of autonomic dysfunction in diabetic and idiopathic gastroparesis. *Gastroenterology Research*.

[41] Liu N., Abell T. (2017). Gastroparesis updates on pathogenesis and management. *Gut and Liver*.

[42] Parameswaran R., Ferrando J., Sigurdsson A. (2006). Gastric bezoar complicating laparoscopic adjustable gastric banding with band slippage. *Obesity Surgery*.

[43] Cortes C., Silva C. (2008). *Revista Medica de Chile*.

[44] Krishnasamy S., Abell T. L. (2018). Diabetic gastroparesis: principles and current trends in management. *Diabetes Therapy*.

[45] Akrami M., Sasani M. R. (2016). Dietary habits affect quality of life: bowel obstruction caused by phytobezoar. *Iranian Journal of Public Health*.

[46] Hsu H. H., Grove W. E., Mindulzun R., Knauer C. M. (1992). Gastric bezoar caused by lecithin: an unusual complication of health faddism. *The American Journal of Gastroenterology*.

[47] Mazid M. A. (2016). Medication bezoar causing acute gastric outlet obstruction: a case report. *Journal of Bangladesh College of Physicians and Surgeons*.

[48] Jain S. A., Agarwal L., Khyalia A., Chandolia P., Kaknale H. (2018). Pharmacobezoar—a rare case presented as gastric outlet obstruction. *Journal of Surgical Case Reports*.

[49] Tabaac B. J., Tabaac V. (2015). Pica patient, status post gastric bypass, improves with change in medication regimen. *Therapeutic Advances in Psychopharmacology*.

[50] Lin X., Mao Y., Qi Q., Zhang C., Tian Y., Chen Y. (2015). Primary systemic amyloidosis initially presenting with digestive symptoms: a case report and review of the literature. *Diagnostic Pathology*.

[51] Appleton E. S., Lee N. A., Ford A. C. (2017). Multiple myeloma presenting in association with gastric phytobezoar. *Clinical Case Reports*.

[52] Lee Y.-I., Lee S.-K. (2016). Endoscopic submucosal dissection of an inverted early gastric cancer-forming false gastric diverticulum. *Clinical Endoscopy*.

[53] Zhu J., Zhu T., Lin Z., Qu Y., Mu D. (2017). Perinatal risk factors for infantile hypertrophic pyloric stenosis: a meta-analysis. *Journal of Pediatric Surgery*.

[54] Purandare S. N., Patil B., Chakane M., Jadhav S. E. (2015). Gallstone bezoar following cholecystogastric fistula: a rare sequelae of cholelithiasis. *Indian Journal of Surgery*.

[55] Pinto D., Carrodeguas L., Soto F. (2006). Gastric bezoar after laparoscopic Roux-en-Y gastric bypass. *Obesity Surgery*.

[56] Ertugrul I., Tardum Tardu A., Tolan K., Kayaalp C., Karagul S., Kirmizi S. (2016). Gastric bezoar after Roux-en-Y gastric bypass for morbid obesity: a case report. *International Journal of Surgery Case Reports*.

[57] Ben-Porat T., Sherf Dagan S., Goldenshluger A., Yuval J. B., Elazary R. (2016). Gastrointestinal phytobezoar following bariatric surgery: systematic review. *Surgery for Obesity and Related Diseases*.

[58] Okagawa Y., Takada K., Arihara Y., Kato J. (2017). A case of gastric perforation caused by chestnut bezoars. *Nihon Shokakibyo Gakkai Zasshi= The Japanese Journal of Gastro-Enterology*.

[59] Grande G., Manno M., Zulli C. (2016). An alternative endoscopic treatment for massive gastric bezoars: Ho:YAG laser fragmentation. *Endoscopy*.

[60] Tudor E. C. G., Clark M. C. (2013). Laparoscopic-assisted removal of gastric trichobezoar; a novel technique to reduce operative complications and time. *Journal of Pediatric Surgery*.

[61] Dai Q., Jiang F. (2018). A huge gastric bezoar treated by traditional Chinese medicine purgative: a case report. *Medicine*.

